# Prevalence and Incidence of Osteoarthritis: A Population-Based Retrospective Cohort Study

**DOI:** 10.3390/jcm10184282

**Published:** 2021-09-21

**Authors:** Rola Hamood, Matanya Tirosh, Noga Fallach, Gabriel Chodick, Elon Eisenberg, Omri Lubovsky

**Affiliations:** 1 Medical Affairs Pfizer Inc., Herzliya 4672509, Israel; Rola.Hamood@pfizer.com; 2 Kahn-Sagol-Maccabi Research and Innovation Institute, Maccabi Healthcare Services, Tel Aviv 6812509, Israel; fallach_n@mac.org.il (N.F.); hodik_g@mac.org.il (G.C.); 3 Sackler School of Medicine, Tel Aviv University, Tel Aviv 6997801, Israel; 4 The Ruth and Bruce Rappaport Faculty of Medicine, Technion—Israel Institute of Technology, Rambam Health Care Campus, Haifa 3525433, Israel; e_eisenberg@rambam.health.gov.il; 5 Barzilai Medical Center Ashkelon Israel, Faculty of Health Sciences, Ben-Gurion University of the Negev, Beer-Sheva 8410501, Israel; omril@bmc.gov.il

**Keywords:** osteoarthritis, prevalence, incidence, trends, Middle East

## Abstract

While trends data of osteoarthritis (OA) are accumulating, primarily from Western Europe and the US, a gap persists in the knowledge of OA epidemiology in Middle Eastern populations. This study aimed to explore the prevalence, incidence, correlations, and temporal trends of OA in Israel during 2013–2018, using a nationally representative primary care database. On 31 December 2018, a total of 180,126 OA patients were identified, representing a point prevalence of 115.3 per 1000 persons (95% CI, 114.8–115.8 per 1000 persons). Geographically, OA prevalence was not uniformly distributed, with the Southern and Northern peripheral districts having a higher prevalence than the rest of the Israeli regions. OA incidence increased over time from 7.36 per 1000 persons (95% CI 6.21–7.50 per 1000 persons) in 2013 to 8.23 per 1000 persons (95% CI 8.09–8.38 per 1000 persons) in 2017 (*p*-value for trend = 0.02). The incidence was lowest in patients under 60 years (in both sexes) and peaked at 60–70 years. In older ages, the incidence leveled off in men and declined in women. The growing risk of OA warrants a greater attention to timely preventive and therapeutic interventions. Further population-based studies in the Middle East are needed to identify modifiable risk factors for timely preventive and therapeutic interventions.

## 1. Introduction

Osteoarthritis (OA) is one of the most prevalent disabling joint disorders [1,2], involving damage to the joint tissues, including articular cartilage, subchondral bone, and synovium [3]. OA represents a significant public health challenge [4], with notable implications for the individuals affected [5], health-care systems, and substantial socioeconomic costs [6]. Although it may develop in any joint [7], a large proportion of the OA burden is due to hip and knee OA, which, at the end-stage, may lead to joint failure, requiring surgical replacement [1,4,7]. The incidence of OA increases with age, and women have higher rates than men, especially after age 50. A leveling off or decrease in incidence occurs at all joint sites around the age of 80 [7]. The prevalence and incidence of OA across studies varies greatly, depending on the definition used, the population sampled (primary versus tertiary care), and the distribution of OA risk factors, such as age, sex, obesity, and geographical region [7,8]. The Global Burden of Disease systematic reviews, conducted by the World Health Organization in 2010 and 2017, have estimated the incidence, prevalence, and disability associated with OA in global regions and individual countries [1,4]. However, because of their design, these reviews provide only a high-level perspective of disease burden. Besides, disease prevalence can change over time, and these reviews include data from studies dating as far back as 1990. Also, because OA susceptibility is strongly affected by genetic and environmental risk factors [7], the assessment of OA epidemiology in such populations not only can contribute to the understanding of the worldwide burden of the disease but may also shed light on the underlying mechanisms of the disease, essential for providing effective preventive strategies. While trends data of this progressive condition are accumulating, primarily from Western Europe and the US, a gap persists in the knowledge of OA epidemiology in Middle Eastern populations [9]. This study aimed to explore the prevalence, incidence, correlations, and temporal trends of OA in Israel during 2013–2018, using a nationally representative primary care database. Although Israel is geographically located in the Middle East, most Israelis live a lifestyle similar to Western Europeans and North Americans. Therefore, we hypothesized that the OA prevalence and incidence would be comparable to those observed in Western countries.

## 2. Materials and Methods

### 2.1. Data Sources

This retrospective study utilized automated data from Maccabi Healthcare Services (MHS), the second-largest Israeli healthcare maintenance organization in Israel [10], providing full medical care to approximately 25% of the total population (2.6 million members). According to the National Health Insurance Law (1995), all Israeli citizens are mandated to be insured by one of four healthcare maintenance organizations, which are run as not-for-profit organizations and prohibited by law from denying any Israeli resident membership; therefore, all Israeli subpopulations are represented in the database. Furthermore, the age and sex distribution of MHS members is similar to that of the general population (median age among MHS members is 31 years; in the general Israeli population: 33 years [11]). The MHS electronic databases capture detailed information on demographics, medical encounters, pharmacy claims, diagnoses, procedures, and other data linked by unique patient identifiers. MHS uses *International Classification of Diseases*, Ninth Revision, Clinical Modification (ICD-9-CM) coding systems, as well as self-developed coding systems, to provide more granular diagnostic information beyond the ICD codes. The data are compiled into a centralized data warehouse from electronic medical records from primary care and specialist clinics, hospitals, pharmacies, and laboratories. Members’ retention rate in Maccabi is very high (99% per year), and therefore, during the two recent decades, over 80% of the study population have been continuously insured by MHS since birth.

### 2.2. Study Population

The study participants included all MHS members, aged ≥18 years, diagnosed with OA (ICD-9-CM diagnosis code 715.x), and continuously insured in MHS during the study period (from 1 January 2013 until 31 December 2018 (inclusive)).

### 2.3. Data Collection

We used the MHS diagnoses databases to obtain information on OA index date, defined as the date of the first diagnosis of OA recorded in the database. Supplementary information obtained from the MHS administrative databases included: age at OA diagnosis, sex, region of residence, weight [kg], body mass index (BMI) [kg/m^2^] in 2018, comorbid conditions (defined by MHS registries or corresponding ICD-9 diagnosis codes) documented between 2013 and 2018 at age ≥18 years old, and smoking (ever/never). Region of residence included five districts that were classified as core (Center, Sharon, Jerusalem, and Shfela districts) and peripheral regions (North and South districts).

#### 2.3.1. Estimation of Incidence and Prevalence

The annual incidence for OA in each calendar year, from 2013 to 2017, was defined as the number of new OA incident cases between 1 January and 31 December, divided by the total MHS population, aged ≥18, at the midpoint for that year, based on the following equation:

$$Incidence=\frac{Annual\;number\;of\;new\;OA\;cases}{Mid-period\;population}$$

The point prevalence of OA was calculated by dividing the number of subjects ever diagnosed with OA, at a specific point in time, by the total number of MHS members at the same time point, based on the following equation:

$$Point\;prevalence=\frac{Number\;of\;existing\;OA\;cases\;at\;a\;specified\;time\;point}{Total\;MHS\;members\;at\;the\;same\;time\;point}$$

#### 2.3.2. Ethics

Maccabi’s Ethics Committee approved the study protocol and conduct and the publication of its results (approval #0100-19-MHS). As this non-interventional administrative study involves anonymized structured data, which according to applicable legal requirements does not contain data subject to privacy laws, obtaining informed consent from patients was waived.

### 2.4. Statistical Analysis

Descriptive statistics of patient characteristics are reported as frequency (*n*, %) for categorical variables and mean and standard deviation (SD) for continuous variables. Total age- and sex-specific proportions of patients diagnosed with OA, for each year from 2013 to 2017, were estimated and presented per 1000 persons. OA point prevalence in 2018 was assessed and stratified by age, sex, and district. Crude prevalence ratios, with respective confidence intervals, were estimated with univariate Poisson regressions. Temporal trends of OA incidence and prevalence over the study period (2013–2017) were evaluated using univariate linear regression. The Kruskal–Wallis test was used to assess whether age, sex, and district groups differed in BMI. Statistical significance was defined as a two-tailed *p*-value < 0.05. All analyses were performed using SAS© software, Version 9.4 (SAS Institute Inc., Cary, NC, USA).

## 3. Results

### 3.1. Description of the OA Cohort

By 31 December 2018, the total number of OA prevalent cases identified was 180,126, yielding a point prevalence of 115.3 per 1000 persons (95% CI, 114.8–115.8 per 1000 persons). Table 1 details the OA study population characteristics in 2018. The mean time elapsed since OA diagnosis was 9.0 years (SD, 5.9), with more than half of cases being diagnosed with OA for at least eight years. The majority of patients with OA (76%) were 60 years or older. About two-thirds were women, 38% resided in the northern and southern peripheries, and most patients were never smokers. The mean weight of OA patients was 78.8 kg (SD, 17.2), corresponding to a BMI of 29.5 kg/m^2^ (SD, 6.2). A minority of OA patients had another concomitant rheumatic disease (e.g., rheumatoid arthritis and ankylosing spondylitis).

The mean BMI of OA patients was positively associated with age (up to age 59 years) and female sex (Table 2). A significant variation in BMI was observed across residence areas, with higher means observed in OA patients residing in the Southern and Northern peripheral districts.

### 3.2. OA Prevalence Temporal Trends and Correlates

**Figure 1:**
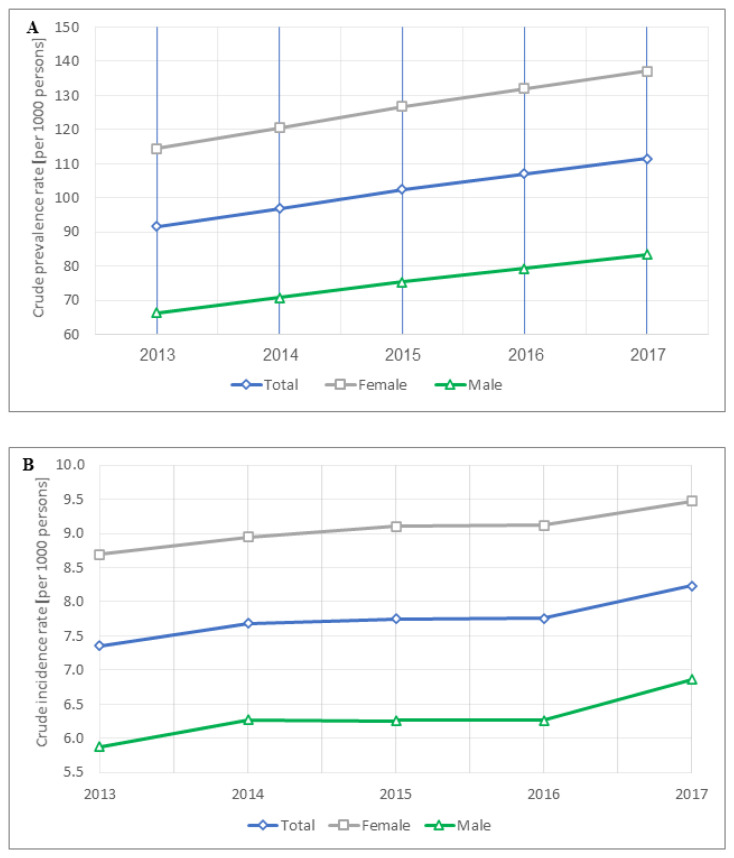
Trends for OA prevalence (**A**) and incidence (**B**) between 2013 and 2017, by sex.

OA prevalence increased monotonically from 2013 to 2017 (*p*-value for trend <0.001). The overall prevalence of OA in 2017 increased to 111.6 per 1000 persons (95% CI, 111.0–112.1 per 1000 persons) from 91.6 per 1000 persons in 2013 (95% CI, 91.1–92.1 per 1000 persons), a 22% increase in prevalence over this period (Figure 1A and Supplementary Table S1). OA prevalence rose in both men and women across the years but was higher in women (*p*-value for trend <0.001 in both sexes). The average annual percentage change was 5.05% for any OA, whereas among men it was 5.89% and, in women, a 4.63% change each year.

**Figure 2:**
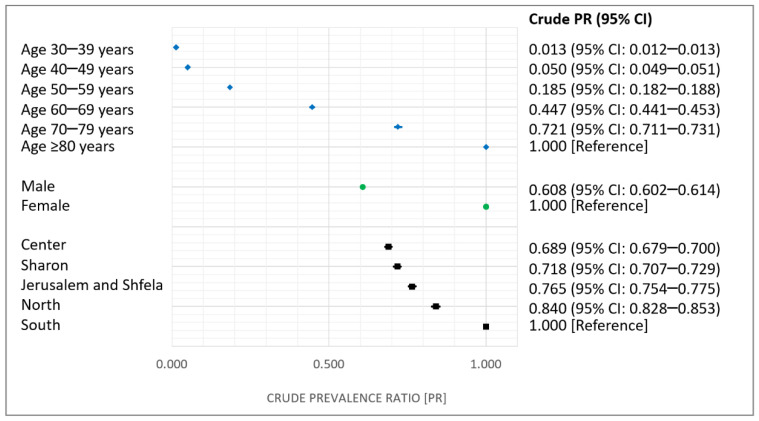
OA prevalence ratios in 2018, by age, sex, and district.

OA crude prevalence ratios and respective 95% CI in 2018, according to age, sex, and district, are shown in Figure 2. A significant positive relationship was observed between age and OA prevalence and followed a dose-response gradient pattern. The crude prevalence of OA in men was significantly lower than that in women (by 39%). OA prevalence varied from one region to another within Israel. The Southern and Northern peripheral districts had the highest OA prevalence of 146.5 per 1000 persons (95% CI, 145.0–148.1 per 1000 persons) and 123.2 per 1000 persons (95% CI, 121.9–124.5 per 1000 persons). The lowest prevalence proportions were observed in the Central and Sharon core districts (101.0 (95% CI, 99.9–102.0) and 105.2 (95% CI, 104.0–106.3) per 1000 persons, respectively). Compared to the southern district, OA prevalence was lower in all other regions, with prevalence ratio reductions ranging between 16% and 31%.

### 3.3. OA Incidence Temporal Trends

**Figure 3:**
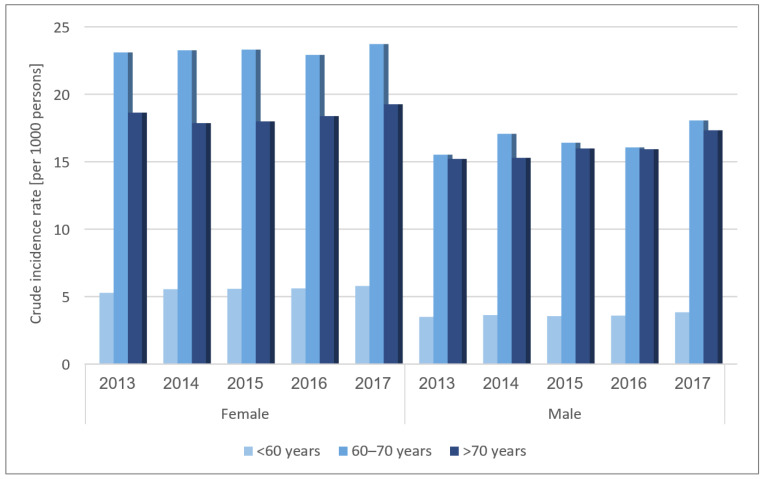
Trends for OA incidence between 2013 and 2017, by age and sex.

During 2013–2017, there were 54,876 incident OA cases. OA incidence increased over time during the study period, changing from 7.36 per 1000 persons (95% CI 6.21–7.50 per 1000 persons) in 2013 to 8.23 per 1000 persons (95% CI 8.09–8.38 per 1000 persons) in 2017 (*p*-value for trend = 0.0245). As with the OA prevalence pattern, OA incidence rose in both men and women (*p*-value for trend = 0.051 and 0.008 among men and women, respectively) but was lower in men throughout the study period. The average annual percentage change was 2.87%, more pronounced in men than women (4.0% versus 2.18%), although the yearly change in men slightly attenuated during 2014–2016 (Figure 1B). Trends for OA incidence between 2013 and 2017, stratified by sex and age, are shown in Figure 3 and Supplementary Table S2. The incidence was lowest in OA patients aged less than 60 years in both sexes, which peaked at 60–70 years, then leveled off in men and declined in women after age 70, a sustaining pattern over the study period. Absolute differences in incidence between both sexes were most prominent among patients aged 60–70, with an average annual change of 6.64 per 1000 persons. However, relative gender differences were more pronounced in patients aged less than 60 years than in the 60–70-year group (average annual percent change of 54.28% and 38.09%, respectively).

## 4. Discussion

With a study population of over 180,000 OA patients, the present study is one the largest in the Middle East to investigate current OA prevalence and incidence trends [9,12,13,14]. Israel may represent a peculiar sample of a developed country, populated with a large diversity of populations from the Middle East, North Africa, Europe, and North America.

### 4.1. OA Prevalence

The crude point prevalence of nearly 12% in our study falls well within the global prevalence range reported in other population-based studies from Europe (10–17%) [15] and North America (12–21%) [15] but tends to be higher than estimates in South America (2–4%) [15] and lower than those in Asia, Africa, and Middle Eastern countries (16–23% [15], 17–25% [15], and 17–29% [12,16], respectively). The observed similarity of OA prevalence in Israel to Western European countries, more than to geographically closer Middle Eastern countries, may be attributed to genetic and environmental factors, as most Israeli embrace a western lifestyle, but also to substantial differences in study population and design, case definition, and database quality [17,18]. We observed a 22% increase in OA prevalence from 2013 to 2017, supporting existing epidemiological evidence, showing an overall upward trend [4,17], although differences do exist. Globally, OA prevalence has increased by 8.5–9.3% from 1990–2017 [4,19]. At the regional level, the percentage change in age-standardized prevalence between 1990 and 2017 was 22.5% in North America, similar to our finding, 7.2% in Western Europe, 8.4% in Central Asia, and 12.8% in North Africa and the Middle East [4]. The observed increase in OA prevalence may reflect an ageing population, increase in risk factors leading to OA [8,20], raised awareness of OA, and progress in detection over the past decade. Regardless of the underlying cause, a constant increase in OA temporal trends suggests that OA’s attributable burden will grow over time, which supports the recognition of OA as one of the leading causes of years lived with disability globally [21].

### 4.2. OA Incidence

We demonstrated a 12% increase in the incidence of OA during the study period, consistent with findings from other studies [4,22,23]. According to the Global Burden of Disease Study 2017 [4], the percentage change in aged-standardized rates between 1990 and 2017 worldwide was 8.2%, 8.0% in Western Europe, 28.2% in North America, 8% in Central Asia, and 12% in North Africa and the Middle East [4]. In contrast, some recent investigations have shown an overall slow decline or no change in incidence rates over time [17,24]. Explanations proposed include improvement in documentation of OA diagnosis codes by moving from ‘unspecified’ OA to a joint-specific coding scheme and physician inclination to report symptoms, rather than a specific diagnosis [17]. The discrepancies in OA incidence trends across studies could also be related to the particular OA definition adopted (self-reported, symptomatic, and radiographic [6]), the length of run-in period use to rule out prevalent cases, the capture and linkage of hospital data, other databases, and population structure [24].

Annually, on average, crude incidence rose by about 2.2–4.0%, in our study, for women and men. Rahman et al. reported an annual relative change of 2.5–3.3% in OA incidence for the period 1991–2009 among persons aged 18 or more in the Province of British Columbia [22]. A positive trend in the incidence of knee OA was observed in a registry-based study from Belgium, between 2006 and 2015, with an annual percentage change of 1.9% [25]. Analysis of a secondary care database from Sweden revealed that population growth and ageing accounted for about one-third of the observed increases in the absolute number of OA hospitalizations, between 1998–2000 and 2013–2015, which suggests that these factors do not fully explain the increased incidence of the disease [26]. The growth of the age stratum, ≥70 years, in our study was not dramatic throughout the study period (8.2% of total MHS members in 2013 to 9.2% in 2017), suggesting that population ageing cannot entirely explain the demonstrated temporal increase in OA incidence. Put differently, OA should not be considered an inevitable consequence of ageing, for there are modifiable preventable factors that may play a role, such as obesity and joint injury [15,26].

### 4.3. OA Correlates

Several demographic factors were associated with OA prevalence in the present study, including age, sex, and region of residence. In line with other reports [20,27], we found a positive association between age and OA incidence, peaking around age 60–70 and leveling off after age 70. Other studies in the UK and Spain, in contrast, found the highest incidence rate of OA among people aged 75–80 years [17,28]. The suspected mechanism leading to joint damage is poorly understood but is probably multifactorial, involving numerous individual factors, such as oxidative damage, thinning of cartilage, sarcopenia, and a reduction in proprioception [7,29].

Congruent with findings from previous studies [4,15,17,21,24], an upward trend was observed in both sexes across the years, but the OA burden was consistently higher in women among all age groups. A large meta-analysis provided evidence to support sex differences in prevalent and incident OA and found that women had more severe radiographic knee OA than men, particularly following menopause [30]. The increase in OA incidence at the time of menopause has led to unestablished hypotheses, regarding the role of estrogen in OA [31]. Sex disparities may also be related to genetics and differences in bone strength, alignment, ligament laxity, pregnancy, and neuromuscular strength [31,32]. We observed that although women had a higher prevalence and incidence of osteoarthritis than men, the relative increase was more prominent in men, in alignment with findings by Spitaels and colleagues [25], but contrasted to Swain et al. who observed a steeper change in annual prevalence rates in women [17]. The exact reason for the differences in findings between studies is unclear, and further investigations in other databases, using comparable methods, are warranted.

We demonstrated geographic variation in the distribution of OA prevalence across districts, with the peripheral rural regions having a higher OA burden than the urban communities. The COPCORD studies conducted in India, Bangladesh, and Pakistan looked specifically into differences between rural and urban populations and reported a higher prevalence in rural communities [33]. The regional variation could reflect differences in practice areas or distribution of OA risk factors, including obesity, physical overload, socioeconomic conditions, lifestyles, and health-seeking behaviors [17,26]. Indeed, higher OA prevalence in the peripheral regions, in the present study, matched the BMI distribution. Obesity is one of the strongest and best-established OA risk factors [7,27]. Silverwood et al. estimated that in patients with new-onset knee pain, 24.6% of cases were related to overweight or obesity [34]. The Framingham study estimated that weight reduction by 5 kg decreased the risk of developing knee OA by 50% [35].

All in all, the increase in OA burden, with advanced age and the epidemic of overweight and obesity, translates into a substantial increase in costs of patient care, home care costs, work loss, absenteeism, and premature retirement [4,8]. Women and those residing in peripheral regions may most be affected; hence, they should be targeted in a prevention and management program [4].

### 4.4. Strengths and Limitations

This large and population-representative study has produced an updated incidence and enabled the comprehensive evaluation of temporal trends in OA over a relatively extended contemporary period. However, certain limitations of this work should be acknowledged. Firstly, aside from the obvious drawbacks of a retrospective design that does not allow us to draw proper inferences on causality, we relied on the routinely collected administrative ICD-9-CM codes, based on physician diagnoses for case definition and without radiographic verification. Concordance between symptoms and radiographic OA is variable and often low, depending on the assessed joint site [36]. Therefore, the possibility of OA misdiagnosis and coding errors cannot be ruled out. Secondly, we used unspecified OA coding for estimating prevalence and incidence. However, there is a wide variation in OA morbidity measures at individual joint sites. For example, in a UK study, no change in incidence trends during 1997–2017 was observed for ankle, foot, wrist, and hand sites, while OA at the knee and hip showed a slightly increasing trend [17]. In MHS databases, the OA site was available for only 861 OA patients (0.5%). If anything, such unspecified reporting could have resulted in an underestimation of the OA burden, as multiple joint involvements could be recorded as one OA event. Thirdly, we could not account for other OA predictors, including occupational history, genetics, diet, or other patient characteristics from the available data. Fourthly, we used crude estimates, instead of standardized estimates. Accordingly, gender and district differences may be confounded by the age composition of the population. Therefore, caution should be taken when interpreting gender and geographical disparities. Lastly, the cohort reflects OA patients in one of four health care maintenance organizations in Israel, thereby limiting the extrapolation of the prevalence and incidence trends to other settings.

## 5. Conclusions

One in seven of either Israelis in peripheral regions or women aged 18 years or more in MHS has a clinical diagnosis of OA in 2018. OA is likely to increase with an ageing population and an obesity pandemic. Women are more affected and burdened by OA than men. Our findings call for improvement in the registration of OA diagnosis codes in the outpatient setting if a more accurate picture of the OA burden is to be achieved. Further prospective population-based studies in the Middle East are warranted to identify modifiable risk factors for timely intervention.

## Figures and Tables

**Table 1:** Characteristics of the study population in 2018.

Characteristic	OA Patients *n* = 180,126 (%)
Mean age at OA diagnosis, years (SD)	58.5 ± 11.9
Age distribution, years	
18–29	637 (0.35)
30–39	2255 (1.25)
40–49	10,183 (5.65)
50–59	29,467 (16.36)
60–69	53,497 (29.70)
70–79	49,795 (27.64)
≥80	34,292 (19.04)
Sex	
Females	115,856 (64.32)
Males	64,270 (35.68)
District	
Center	34,534 (19.17)
Jerusalem and Shfela	43,025 (23.89)
North	34,848 (19.35)
Sharon	33,835 (18.78)
South	33,884 (18.81)
Mean time elapsed since OA diagnosis, years	9.0 ± 5.9
Time elapsed since OA diagnosis distribution, years	
<4	41,455 (23.01)
4–7	40,161 (22.30)
8–13	49,862 (27.68)
≥14	48,648 (27.01)
Mean BMI, kg/m^2^ (SD)	29.5 ± 6.2
Mean weight, kg (SD)	78.8 ± 17.2
Smoking	
Ever	43,894 (24.37)
Never	126,552 (70.26)
Unknown	9680 (5.37)
Comorbid conditions between 2013–2018	
Ischemic heart disease	21,359 (11.86)
Cancer	36,212 (20.10)
Diabetes	47,229 (26.22)
Hypertension	105,252 (58.43)
Osteoporosis	41,260 (22.91)
Chronic kidney disease	61,490 (34.14)
Rheumatoid arthritis	4054 (2.25)
Inflammatory bowel disease	1959 (1.09)
Psoriasis	9562 (5.31)
Psoriatic arthritis	1628 (0.90)
Ankylosing spondylitis	700 (0.39)
Fibromyalgia	31,498 (17.49)
Depression/Anxiety	21,455 (11.91)

Abbreviations: OA, osteoarthritis; SD, standard deviation; BMI, body mass index. Data are presented as no. (%) unless otherwise noted; percentages may not sum to 100, due to rounding.

**Table 2:** Body mass index of 180,126 OA patients in 2018, by age, gender, and district.

Characteristic	Mean BMI (kg/m^2^) (SD)	*p*
Age, years		
18–29	24.7 (4.9)	<0.0001
30–39	27.3 (5.8)	
40–49	29.2 (5.7)	
50–59	29.7 (5.5)	
60–69	29.6 (5.2)	
70–79	29.3 (5.0)	
≥80	28.6 (4.9)	
Sex		
Females	29.5 (5.5)	<0.0001
Males	28.9 (4.6)	
District		
Center	28.4 (5.0)	<0.0001
Sharon	28.8 (5.0)	
Jerusalem and Shfela	29.4 (5.1)	
North	29.7 (5.2)	
South	30.0 (5.2)	

Abbreviations: OA, osteoarthritis; SD, standard deviation; BMI, body mass index.

## Data Availability

Data sharing not applicable.

## Supplementary Materials

The following are available online at https://www.mdpi.com/article/10.3390/jcm10184282/s1, Table S1: Trends for OA prevalence between 2013 and 2017, by sex; Table S2: Trends for OA incidence between 2013 and 2017, by age and sex.
