# Supplementary material for: Prevalence and Incidence of Osteoarthritis: A Population-Based Retrospective Cohort Study
Source: J Clin Med. 2021 Sep 21;10(18):4282. doi: 10.3390/jcm10184282 (PMC8468886; doi:10.3390/jcm10184282)
Supplement: Supplementary file 1 [file jcm-10-04282-s001.zip › jcm-1330983-supplementary.pdf]

Supplementary material

Table S1. Trends for OA prevalence between 2013 and 2017, by sex.

Year	Total			Female			Male		
	Cases	MHS population	Prevalence per 1000 persons	Cases	MHS population	Prevalence per 1000 persons	Cases	MHS population	Prevalence per 1000 persons
2013	122,481	1,337,111	91.6	80,440	703,263	114.4	42,041	633,848	66.3
2014	133,014	1,372,126	96.9	86,896	721,432	120.4	46,118	650,694	70.9
2015	143,900	1,404,452	102.5	93,612	738,194	126.8	50,288	666,258	75.5
2016	155,155	1,450,360	107.0	100,560	762,024	132.0	54,595	688,336	79.3
2017	167,519	1,501,612	111.6	108,027	788,134	137.1	59,492	713,478	83.4

Abbreviations: OA, osteoarthritis; MHS, Maccabi healthcare services.

Table S2. Trends for OA incidence between 2013 and 2017, by age and sex.

	Year	Total			Female			Male		
		Cases	MHS population	Incidence per 1000 persons	Cases	MHS population	Incidence per 1000 persons	Cases	MHS population	Incidence per 1000 persons
Total patients	2013	9,838	1,337,111	7.36	6,108	703,263	8.69	3,730	633,848	5.88
	2014	10,533	1,372,126	7.68	6,456	721,432	8.95	4,077	650,694	6.27
	2015	10,886	1,404,452	7.75	6,716	738,194	9.10	4,170	666,258	6.26
	2016	11,255	1,450,360	7.76	6,948	762,024	9.12	4,307	688,336	6.26
	2017	12,364	1,501,612	8.23	7,467	788,134	9.47	4,897	713,478	6.86
<60 years	2013	4,696	1,061,165	4.43	2,918	552,656	5.28	1,778	508,509	3.50
	2014	5,007	1,080,612	4.63	3,124	562,477	5.55	1,883	518,135	3.63
	2015	5,059	1,097,688	4.61	3,187	570,871	5.58	1,872	526,817	3.55
	2016	5,193	1,118,620	4.64	3,264	581,578	5.61	1,929	537,042	3.59
	2017	5,579	1,149,626	4.85	3,459	597,769	5.79	2,120	551,857	3.84
60-70 years	2013	3,269	167,409	19.53	2,044	88,497	23.10	1,225	78,912	15.52
	2014	3,630	178,387	20.35	2,196	94,400	23.26	1,434	83,987	17.07
	2015	3,796	189,238	20.06	2,334	100,147	23.31	1,462	89,091	16.41
	2016	3,882	197,137	19.69	2,392	104,383	22.92	1,490	92,754	16.06
	2017	4,248	201,821	21.05	2,530	106,677	23.72	1,718	95,144	18.06
>70 years	2013	1,873	109,278	17.14	1,146	61,472	18.64	727	47,806	15.21
	2014	1,896	113,310	16.73	1,136	63,607	17.86	760	49,703	15.29
	2015	2,031	118,733	17.11	1,195	66,415	17.99	836	52,318	15.98
	2016	2,180	126,003	17.30	1,292	70,276	18.38	888	55,727	15.93
	2017	2,537	137,861	18.40	1,478	76,746	19.26	1,059	61,115	17.33

Abbreviations: OA, osteoarthritis; MHS, Maccabi healthcare services.
